# Comparing the Influence of Surgical and Conservative Therapy on Quality of Life in Patients with Early-Stage Medication-Related Osteonecrosis of the Jaw—A Prospective Longitudinal Study

**DOI:** 10.3390/medicina59020277

**Published:** 2023-01-31

**Authors:** Thomas Rückschloß, Maximilian Smielowski, Julius Moratin, Gregor Schnug, Maximilian Appel, Philipp Muench, Moritz Bleymehl, Sven Zittel, Michael Engel, Jürgen Hoffmann, Oliver Ristow

**Affiliations:** Department of Oral and Maxillofacial Surgery, University of Heidelberg, Im Neuenheimer Feld 400, D-69120 Heidelberg, Germany

**Keywords:** ARONJ, MRONJ, therapy, management, quality of life, QLQ-C30, QHIP

## Abstract

*Background and Objectives*: The purpose of this study was to evaluate the impact of surgical and conservative, non-surgical treatment on general health-related (QoL) and oral health-related quality of life (OHRQoL) in patients suffering from AAOMS stage I MRONJ. *Materials and Methods:* In the course of this prospective clinical study, QoL and OHRQoL using QLQ-C30 and QHIP G14 questionnaire were longitudinally assessed in N = 174 prospectively enrolled patients with indication of treatment of MRONJ stage I over a period of 12 months. Patients received conservative or surgical treatment. The measurement time points were preoperatively (T0), 12 weeks (T1), 6 months (T2) and 1 year after operation (T3). *Results:* For OHRQoL, no significant (*p* > 0.05) differences were found between both treatment groups for all timepoints (T0–T3). In the surgical treatment group, OHIP scores of T1, T2 and T3 were significantly lower than baseline measures (T0) (T0–T1 (2.99, *p* = 0.024), T0–T2 (5.20, *p* < 0.001), T0–T3 (7.44, *p* < 0.001)). For conservative treatment group OHIP, scores of T2 and T3 were significantly lower than baseline measures (T0) (T0–T2 (9.09, *p* = 0.013), T0–T3 (12.79, *p* < 0.001)). There was no statistically significant effect of time on QLQ-C30 scores in both groups (surgical treatment: F(3, 174) = 1.542, *p* < 0.205, partial η^2^ = 0.026; conservative treatment: F(3, 30) = 0.528, *p* = 0.667, partial η^2^ = 0.050). QLQ-C30 scores turned out to be significantly lower in the non-surgical group at T1 (*p* = 0.036) and T3 (*p* = 0.047) compared to the surgical treatment group. *Conclusions:* Surgical and conservative treatment of MRONJ stage I significantly improves patients’ OHRQoL. Surgical treatment is superior to conservative treatment of MRONJ stage I regarding general QoL. Therefore, surgical treatment of MRONJ stage I should not be omitted for QoL reasons.

## 1. Introduction

Patients with oncological diseases that metastasize to bone and patients with osteoporosis are at increased risk of suffering from skeletal-related events (SREs) such as pathological fracture, radiation or surgery to bone and spinal cord compression [1,2]. These SREs are a major burden on healthcare systems worldwide [3,4], and they severely reduce patients’ quality of life (QoL) [2]. In order to prevent SREs or the advancement of bone metastases in the aforementioned patient populations, antiresorptive medications, particularly bisphosphonates and denosumab, are crucial parts of pharmaceutical therapy. These substances have a positive impact on health-related quality of life (QoL) [5].

In general, antiresorptive medications are reported to have little side effects and are very well tolerated. However, it has been demonstrated recently that a severe side effect of these drugs is medication-related osteonecrosis of the jaw (MRONJ) [6,7,8].

MRONJ can have a complicated path that results in the loss of dental and oral function, as well as a potential large-volume loss of jaw parts, which lowers both general and oral health-related quality of life (OHRQoL) [9]. In addition, a diagnosis of MRONJ—and occasionally merely a suspicion of it—causes the suspension of antiresorptive and oncological therapy. This could have an effect on the underlying pathology and indirectly on patients’ quality of life [10].

Many international professional societies have published guidelines for the management of antiresorptive treated patients and MRONJ [7,11,12,13]. All treatment strategies, but especially those for early stages [14], remain controversial [7,9]. In particular, the American Association of Oral and Maxillofacial Surgeons (AAOMS) suggests non-surgical treatment for early stages in its position paper. These recommendations are based on a clinically driven staging system (stage 0-III) [12].

A growing body of evidence, however, indicates that treating individuals with MRONJ—regardless of the underlying stage—with surgical removal of necrotic bone may be curative [9,15,16,17]. In this context, cure is defined as long-term symptom relief and complete healing of the mucosa (absence of residual bone exposure). 

Retrospective works [18,19] as well as systematic reviews [20] have shown cure rates of 95 to 100% with surgical therapy in the lower and upper jaw. The advantages of surgical therapy are the earliest possible resumption of antiresorptive therapy, a reduction in pain, rapid dental rehabilitation and prevention of progression of necrosis to higher stages [18,19,20].

To improve the treatment and outcomes of patients suffering from MRONJ, a deeper understanding of how the disease and its treatment affect patients’ general and OHRQoL is of the utmost importance. QoL is an important patient-oriented factor, particularly in oncological patients with a palliative overall prognosis. As a result, it is critical to consider QoL when weighing risks and making treatment decisions for these critically ill patients.

The purpose of this study was to determine how MRONJ stage I affects patients’ QoL and OHRQoL, as well as how different therapy regimens affect these outcome measures.

We hypothesize that surgical therapy for MRONJ stage I results in significant improvement in general and OHRQoL compared to conservative therapy. 

## 2. Materials and Methods

### 2.1. Study Design 

We designed a prospective longitudinal study that compares the surgical/operative and non-surgical/conservative treatment outcomes of patients suffering from MRONJ stage I. The investigation was carried out at the Department of Cranio-, Oral- and Maxillofacial Surgery, University of Heidelberg. The study protocol was registered in the German Clinical Trials Register (DRKS00012888).

### 2.2. Patients

All patients with MRONJ stage I who were assigned to our department for treatment were consecutively recruited and checked for eligibility in our specialized consultation hour over a period of 36 months between 2019 and 2021. 

The inclusion criteria for the trial were as follows: (a) patients with not infected medication-related osteonecrosis of the jaw (stage I [14]) with previous or ongoing antiresorptive treatment with bisphosphonates or denosumab; (b) written consent to participation in the trial. The exclusion criteria were as follows: (a) history of head and neck radiation, (b) metastatic bone disease of the maxillofacial region and (c) patients younger than 18. 

### 2.3. Data Collection

All surgical interventions were performed under total anesthesia by pre-established surgeons following the surgical protocol as published previously [16], in adherence to a standardized intra-institutional protocol following the German guidelines for MRONJ [11]. Following the operation, we provided additional follow-up care during our weekly consultation hours.

The non-surgical treatment followed our institutional protocol as published previously [9]. In short, treatment consisted of antimicrobial mouth rinsing by applying 0.2% chlorhexidine solution (GlaxoSmithKline Consumer Healthcare GmbH& Co. KG, Munich, Germany) three times a day and the daily topical application of 1% chlorhexidine gel (GlaxoSmithKline Consumer Healthcare GmbH & Co. KG, Munich, Germany). Manual cleansing of the necrotic region was performed at regular intervals of four weeks. During follow-ups, in cases of spontaneous formation and dissolution of superficial bone sequestra, a surgical removed was performed. With regard to the AAOMS definition of MRONJ stage I as exposed jaw bone or fistula that extends to the bone in asymptomatic patients without signs of infection and lack of symptoms [14], no antibiotics were utilized. 

As soon as MRONJ was diagnosed, the use of bisphosphonates or denosumab was suspended. The decisions on this were always made by the treating oncologists. It must be emphasized that we therefore had no influence on this.

### 2.4. Questionnaires (QLQ-C30, OHIP G49)

At baseline, before MRONJ treatment (T0), 12 weeks (T1), 6 months (T3) and 1 year after treatment (T3), all patients completed the OHIP-G14 and QLQ-C30 questionnaires.

The Oral Health Impact Profile 14 (OHIP-G14) [21,22] is a short-form oral health impact profile based on the longer OHIP-G49 questionnaire [23,24]. In the original OHIP-G49 questionnaire, participants were asked how frequently they had a certain symptom the week before. It consists of 49 items and covers seven domains, namely Functional Limitation (9 items), Physical Pain (9 items), Psychological Discomfort (5 items), Physical Disability (9 items), Psychological Disability (6 items), Social Disability (5 items) and Handicap (6 items). For each of the 49 OHIP questions, subjects rate on an ordinal scale (0 “never”, 1 “hardly ever”, 2 “occasionally”, 3 “fairly often”, 4 “very often”) how frequently they have experienced a specific oral health impact, with a lower index score indicating a better OHRQoL. The most widely used OHIP version nevertheless is the G14 version with 14 items, 2 items for each of the above-mentioned seven domains [22,25]. Recently, John et al. reclassified the OHRQoL. They advise only to compute the domain scores for Physical Disability, Physical Pain, Psychological Discomfort and Handicap scores. These scores should be renamed to Oral Function, Oralfacial Pain, Orofacial Appearance and Psychosocial Impact scores, respectively [25]. The sum of the scores for each item was used to construct the so-called OHIP index score. Lower index scores indicate a better evaluation of OHRQoL. The scores of the items pertaining to each of the domains were added to determine the scores for each domain.

The second questionnaire used was the European Organisation for Research and Treatment of Cancer (EORTC) QLQ-C30 [26]. It is a questionnaire consisting of 30 items resulting in five functional scales (physical, role, cognitive, emotional and social), three symptom scales (fatigue, pain and nausea and vomiting) and a global health and quality-of-life scale. Further, it contains single items assessing common symptoms of patients suffering from cancer, these are dyspnea, appetite loss, sleep disturbance, constipation, diarrhea and financial impact [26]. Since a detailed explanation of the calculation of the QLQ-C30 scores is beyond the scope of this paper, we refer to the corresponding scoring manual [27].

### 2.5. Statistical Analysis

For statistical computations, SPSS version 25.0 was used (SPSS Inc., Chicago, IL, USA). No sample size calculation was performed. Descriptive statistics were used to characterize patient demographics and clinical details. The QoL results were presented in accordance with the recommendations/guidelines of John et al. and the EORTC [24,28]. However, due to the lack of homogeneity of the patient collective, only the QLQ-C30 general health score and OHIP G14 sum score were evaluated exploratively. The Shapiro–Wilk test was used to test for normal distribution. Homogeneity of the error variances was assessed using Levene’s test. Homogeneity of covariances was assessed with Box’s test. A test for sphericity was not used as the collective only consisted of two groups (surgical/non-surgical treatment). To compare quality of life as presented by OHIP G14 and QLC-C30 score between both groups, a mixed-ANOVA model that uses the respective post-intervention value as outcome was computed, together with the baseline value and the treatment group. To investigate differences between the groups at all time points (T0–T3), a standard *t*-test was utilized. As we only included patients with complete datasets, no imputation was required.

## 3. Results

### 3.1. Patient Characteristics

A total of 174 patients fulfilled the inclusion criteria and were included (108 females and 66 male patients). Of these patients, 140 received surgical treatment, while 34 received conservative treatment. Patients’ characteristics are shown in Table 1. 

### 3.2. OHIP

Descriptive statistics for OHIP-14 index scores are provided in Table 2. There was homogeneity of the error variances, as assessed by Levene’s test (*p* > 0.05), as well as homogeneity of covariances, as assessed by Box’s test (*p* > 0.259). The Greenhouse–Geisser adjustment was used to correct for violations of sphericity (Mauchly test: *p* ≤ 0.001). There was no statistically significant interaction between time and group (Greenhouse–Geisser F(2.763, 475.234) = 1.879, *p* = 0.137, partial η^2^ = 0.011). There was a significant main effect for time (Greenhouse–Geisser F(2.763, 475.234) = 26.902, *p* < 0.001, partial η^2^ = 0.135). There was no significant main effect for group, meaning that intervention groups did not differ (F(1, 172) = 0.004, *p* = 0.949, partial η^2^ ≤ 0.001). To assess the between-subject effects, two-sided t-tests were calculated. There was no significant (*p* > 0.05) difference between both treatment groups for all timepoints (T0–T3). To assess the within-subject effects, an ANOVA for each treatment group was calculated. There was a statistically significant effect of time on OHIP scores in both groups (surgical treatment: Greenhouse–Geisser F(2.774, 385.546) = 19.928, *p* < 0.001, partial η^2^ = 0.125; non-surgical treatment: Greenhouse–Geisser F(2.668, 88.048) = 10.526, *p* < 0.001, partial η^2^ = 0.242). According to the Tukey HSD, in the surgical treatment group, OHIP scores of T1, T2 and T3 differed significantly from baseline measures (T0) (T0–T1 (2.99, *p* = 0.024), T0–T2 (5.20, *p* < 0.001), T0–T3 (7.44, *p* < 0.001)). For the non-surgical treatment group, Tukey HSD showed that OHIP scores of T2 and T3 differed significantly from baseline measures (T0) (T0–T2 (9.09, *p* = 0.013), T0–T3 (12.79, *p* < 0.001)). For the other OHIP 14 results (oral function, orofacial pain, orofacial appearance, psychosocial impact score) please refer to Table S1.

### 3.3. QLQ-C30

Please refer to Table 3 for descriptive statistics of QLQ-C30 global health status. There was homogeneity of the error variances, as assessed by Levene’s test (*p* > 0.05), as well as homogeneity of covariances, as assessed by Box’s test (*p* > 0.05). No violations of sphericity were found (Mauchly test: *p* ≤ 0.423). There was no statistically significant interaction between time and group, F(3, 204) = 1.543, *p* = 0.204, partial η^2^ = 0.022. There was no significant main effect for time, F(3, 204) = 0.824, *p* < 0.482, partial η^2^ = 0.012. There was a significant main effect for group, meaning that intervention groups did differ with regard to QLQ C30 scores, F(1, 68) = 4.340, *p* = 0.041, partial η^2^ ≤ 0.060. To assess the between-subject effects, two-sided t-tests were calculated. QLQ-C30 scores turned out to be significantly lower in the non-surgical group at T1 (*p* = 0.036) and T3 (*p* = 0.047) compared to the surgical treatment group. To assess the within-subject effects, an ANOVA for each treatment group was calculated. There was no statistically significant effect of time on QLQ-C30 scores in both groups (surgical treatment: F(3, 174) = 1.542, *p* < 0.205, partial η^2^ = 0.026; non-surgical treatment: F(3, 30) = 0.528, *p* = 0.667, partial η^2^ = 0.050). No post hoc test was calculated. To assess the between-subject effects, two-sided t-tests were calculated. QLQ-C30 scores turned out to be significantly lower in the non-surgical group at T1 (*p* = 0.036) and T3 (*p* = 0.047) compared to the surgical treatment group. For the other QLQ-C30 results (functional scales and symptom scales) please refer to Table S2.

## 4. Discussion

This study’s objective was to assess the effects of surgical and non-surgical treatment on general and OHRQoL in patients suffering from AAOMS stage I MRONJ [12,14].

We were able to show from our studied population that OHRQoL improves significantly over time with conservative as well as surgical therapy for MRONJ stage I. Interestingly, there were no significant differences between the groups. It could be concluded that both surgical and conservative therapy are effective in preventing the development of OHRQoL-affecting symptoms. We will discuss this aspect in the following.

Furthermore, we demonstrated that general QoL in the surgically treated patient group remained relatively constant during the follow-up interval. In contrast, the general QoL of the conservatively treated patients was significantly lower than that of the surgically treated patients at some timepoints (T1 and T3). From this, it could be deduced that surgical treatment is superior to conservative treatment regarding general QoL. This is also discussed in the following.

Especially in recent years, in view of the increasing number of cases and the increasing prescription of antiresorptive drugs, MRONJ has become increasingly present in the clinical routine of oncologists and maxillofacial surgeons. While initially the pathogenesis, diagnosis and therapy of MRONJ were in the foreground of clinical research, in recent years patient-related outcome measures, especially QoL, have increasingly become the focus of scientific efforts. This is reflected in the growing body of literature dealing with the topic.

Two recent systematic reviews [29,30] on this topic identified a total of 12 studies that examined quality of life in MRONJ patients [10,31,32,33,34,35,36,37,38,39,40,41]. Another literature search by our research group identified six more papers [42,43,44,45,46,47]. Ten of the studies assess OHRQoL (using the questionnaires OHIP 14, OHIP 49, QLQ-OH 15, QLQ-HN35) [10,32,33,36,37,39,41,42,43,44], while twelve looked at general health-related QoL, pain or psychological outcome measures (using the questionnaires: EORTC QLQ-C30, SF 12, UWQoL, EQ-5D, Duke health profile, VAS, QLQ-ELD14, SWLS) [31,32,34,35,36,37,38,40,41,42,44,47]. One working group developed and validated a separate QLQ questionnaire specifically for patients with MRONJ [45]. The aforementioned research looked at the impact of MRONJ alone or in combination with various forms of therapy on patients’ QoL.

It has been demonstrated that MRONJ patients typically have a low quality of life and a number of oral symptoms, such as discomfort and speech issues [29]. 

Poorer QoL appeared to be associated with advanced MRONJ stages [30,41]. In their work, Miksad et al. performed a time trade-off (TTO) analysis in which cancer patients suffering from MRONJ were asked whether they would trade the time they would spend in their current health state for their remaining life in perfect health. They showed that patients with a higher stage of MRONJ were more willing to trade their remaining life for better quality of health [41]. Using other questionnaires (OHIP 14, EQ 5D), they were also able to show that the magnitude of the negative QoL effects of ONJ stages 2 and 3 are equivalent to other cancer treatment side effects that influence treatment decisions [41]. The most noticeable change was between stages one and two [41]. In a prospective clinical trial in 36 patients with MRONJ, Winter et al. showed that MRONJ defect size has a significant effect on satisfaction with life [42]. Patients with larger defects were significantly less satisfied with life. Interestingly, Winter et al. did not find significant differences between MRONJ stages [42]. It is certainly debatable whether the current AAOMS staging [12] correctly reflects the defect size. Recent work has shown that this is not the case [48].

An important aspect to keep in mind when considering the quality of life of MRONJ patients is that they are an extremely complex and sometimes heterogeneous patient population. Thus, antiresorptive drugs are used not only to treat metastatic oncological disease but also to prevent metastasis or treat osteoporosis. As such, oncological and osteoporotic patients, whether or not they suffer from MRONJ, differ in terms of their quality of life. It is, in fact, difficult to distinguish whether a reduction in quality of life is caused by the underlying disease or MRONJ [29,30]. In studies of cancer patients receiving palliative care, two-thirds of these patients were found to suffer from oral complaints, such as xerostomia and taste changes, which reduced their quality of life. This is because other oncological drugs, such as some chemotherapeutic drugs, can also affect oral health [49]. Most currently existing studies on our topic examine both oncological and osteoporotic patients. Few examine purely oncological [32,33,40,41,43,44] or osteoporotic patients [36].

Especially in studies in which only the general QoL is measured, distinguishing between diseases is important. For example, Oteri et al. showed different baseline values in general QoL in patients with osteoporosis and those with cancer [37]. 

In the present work, we evaluated the influence of conservative and surgical treatment on stage I MRONJ patients’ QoL. Unfortunately, the available studies [10,31,34,36,37,38,39,42,44,46] on this subject differ greatly with regard to the methods of treatment, indications for the respective therapy and the stages of MRONJ. Most authors examined the influence of conservative or surgical therapy according to the recommendation of the AAOMS from 2014 [14]. These AAOMS recommendations suggest generally non-surgical treatment for stages 1 and 2 and performing surgical debridement/resection of necrotic bone only for stage 3 MRONJ patients [12,14,50]. As a result, the existing studies on our topic performed surgical interventions mainly in stage 3 patients, and thus there are few data on early-stage surgical treatment. However, there is growing evidence that surgical removal of necrotic bone is not only curative in patients with all stages of MRONJ [16]. Furthermore, comparative studies have even demonstrated the superiority of surgical therapy compared to nonsurgical treatment [7,15,16,51]. The recommendation for early surgical therapy has therefore already been included in many international guidelines [7,11,13]. Some studies also deal with novel therapeutic techniques, for example the use of adjunctive hyperbaric oxygen (HBO) [31] or the application of platelet-rich fibrin (PRF) [38,52]. These differences in treatment approaches therefore make the existing data difficult to interpret. However, in principle, existing studies agree that therapy, whether surgical or conservative, improves or at least does not worsen patients’ quality of life [29,30]. Comparing conservative and surgical treatment, resection of necrosis seems to be superior with regard to an improvement of symptoms and QoL [37]. To the best of our knowledge, the present study is the first to evaluate surgical and conservative therapy in AAOMS stage I patients with regard to general and OHRQoL. 

One of the pillars of the AAOMS’ argumentation, until the update in 2022 on the question of why conservative rather than surgical therapy should be used in MRONJ stages I and II, was that the patient’s quality of life must be maintained [12,14]. Although the AAOMS qualify their management recommendation somewhat in that they have a derived shared-decision model depending on patient’s general condition and in an interdisciplinary exchange, they still maintain that conservative therapy is sensible, especially for the early stages, and should always be carried out in consideration of the quality of life.

From their point of view, stage I in particular can be managed with local conservative wound care. This recommendation is completely at odds with an increasing quantity of data, which show that with earlier surgery, not only is bone and dental loss reduced, but the prognosis is also improved. This leads to earlier dental rehabilitation and thus indirectly to an improved QoL.

Furthermore, as we have been able to show and as other studies have demonstrated, conservative therapy of MRONJ stage I and II can bring about an improvement in OHRQoL. This does not apply to the general QoL, however. It is therefore questionable why the AAOMS continues to adhere to its recommendations. 

The question arises why surgical treatment is superior to conservative therapy in terms of maintaining general QoL. An important point here is certainly the question of whether patients had a drug holiday of antiresorptive treatment. A drug holiday could lead to a progression of the underlying disease as well as SREs. These are all associated with high mortality [53] and reduction in QoL. It is important to mention that all our patients had a drug holiday. In all cases, it had previously been initialized by the attending oncologist or osteologist. Consequently, we were not involved in the decision. However, in our surgically treated patients, we were able to continue antiresorptive therapy in consultation with the oncologists/osteologists after wound healing was complete.

The main limitation of this study is certainly that there was no disease-free control group to precisely evaluate the difference in QoL between patients affected by ONJ and disease-free patients. In addition, similar to other study groups, our patient population was not homogeneous, as both oncological and osteoporotic patients were studied. 

Furthermore, both study arms differ in terms of underlying diseases and number of patients. This inhomogeneity is certainly due to the study design. We designed a prospective longitudinal study that compares the surgical and conservative treatment outcomes of patients suffering from MRONJ stage I. As is known from the literature and has now been included in several guidelines, surgical therapy is superior to conservative therapy in terms of therapeutic success. Based on the available knowledge, it does not seem ethically justifiable to set up a RCT. For this reason, the allocation of patients was based on informed consent. This certainly introduces a selection bias, as patients with severely compromised health are more likely to opt for conservative therapy rather than surgery. Because of the different oncological diseases, a subgroup analysis was not useful in statistical terms. Furthermore, no analysis of pain status was included in our study. Pain is a factor that directly affects patients’ QoL. Another factor that was not surveyed is smoking habits and secondary diseases such as diabetes, which have a negative impact on wound healing in MRONJ patients.

Although our study examines the largest patient population with MRONJ in terms of QoL, further studies with larger and more homogeneous patient populations are needed.

Another limitation is certainly that it was not documented how many teeth were lost due to MRONJ therapy and what the subsequent prosthetic restoration was. Winter et al., for example, showed that the need for prosthodontics rehabilitation after MRONJ treatment reduced the OHRQoL at baseline and follow-up even more than MRONJ without the need for prosthodontics alone. 

Based on our findings, we recommend an early and consequent surgical treatment of MRONJ given that patients’ general conditions allow it. Oral examination should be scheduled during antiresorptive treatment for early detection of MRONJ. This may minimize the negative effects of MRONJ on QoL and OHRQoL.

## 5. Conclusions

The following conclusions can be made given the constraints of this study:

- Surgical and conservative treatment of MRONJ stage I significantly improves patients’ OHRQoL.
- Surgical treatment is superior to conservative treatment of MRONJ stage I regarding general QoL.

Therefore, surgical treatment of MRONJ stage I should not be omitted, especially since the earlier the operation is performed, the fewer bones and teeth have to be removed, the sooner the resumption of oncological and osteological therapy and, last but not least, the easier and faster the dental rehabilitation.

## Figures and Tables

**Table 1:** Descriptive statistics.

	Surgical Treatment		Conservative Treatment	
	N	%	N	%
Gender				
Female	86	61.4	22	64.7
Male	54	38.6	12	35.3
Age (years)				
Mean	70.3		69.6	
SD	13.2		11.3	
Cancer types				
Breast	38	27.1	16	47.1
Prostate	29	20.7	5	14.7
Renal	6	4.3	3	8.8
Multiple myeloma	19	13.6	10	29.4
Osteoporosis	36	25.7	0	0
Others	12	8.6	0	0
Bone metastasis				
No	36	25.7	0	0
Yes	85	60.7	24	70.6
Multiple Myeloma	19	13.6	10	29.4
Antiresorptive Treatment				
Bisphosphonates	105	75.0	27	79.4
Denosumab	28	20.0	6	17.6
Both	7	5.0	1	2.9
Duration antiresorptive treatment (months)				
Mean	53.7		55.8	
SD	41.3		42.4	

**Table 2:** Descriptive statistics OHIP 14 index scores (*p* < 0.05. Equality of variance assumed, two-tailed *t*-test).

	Surgical Treatment		Non-Surgical Treatment		*p*-Value for Group Difference, Two-Tailed
Timepoint	Mean	SD	Mean	SD	
T0	14.1	11.6	17.1	11.8	0.175
T1	11.1	11.5	11.6	11.3	0.808
T2	8.9	11.5	8.1	10.9	0.698
T3	6.7	10.7	4.4	8.2	0.241

**Table 3:** Descriptive statistics QLQ-C30 global health status (* *p* < 0.05. Equality of variance assumed, two-tailed *t*-test).

	Surgical Treatment		Non-Surgical Treatment		*p*-Value for Group Difference, Two-Tailed
Timepoint	Mean	SD	Mean	SD	
T0	55.1	22.0	49.0	21.7	0.159
T1	55.8	26.1	44.3	23.1	0.036 *
T2	50.5	25.6	40.6	23.1	0.095
T3	58.6	17.5	46.5	26.7	0.047 *

## Data Availability

The data presented in this study are available in justified cases on request from the corresponding author.

## Supplementary Materials

The following supporting information can be downloaded at: https://www.mdpi.com/article/10.3390/medicina59020277/s1, Table S1: Comparison of the surgically and the non-surgically treated groups over the timepoints T0, T1, T2 and T3 with regard to OHIP 14 scores; Table S2: Comparison of the surgically and the non-surgically treated group over the timepoints T0, T1, T2 and T3 with regard to QLQ-C30 scores.
