# Supplementary material for: Comparing the Influence of Surgical and Conservative Therapy on Quality of Life in Patients with Early-Stage Medication-Related Osteonecrosis of the Jaw—A Prospective Longitudinal Study
Source: Medicina (Kaunas). 2023 Jan 31;59(2):277. doi: 10.3390/medicina59020277 (PMC9966476; doi:10.3390/medicina59020277)
Supplement: Supplementary file 1 [file medicina-59-00277-s001.zip › medicina-2122345-supplementary.pdf]

Table S1. Comparison of the surgically and the non-surgically treated groups over the timepoints T0, T1, T2 and T3 with regard to OHIP 14 scores.

		Surgical treatment		Non-surgical treatment		*p*-value for group difference, two-tailed
	Timepoint	Mean	SD	Mean	SD	
Index score	T0	14.1	11.6	17.1	11.8	0.175
	T1	11.1	11.5	11.6	11.3	0.808
	T2	8.9	11.5	8.1	10.9	0.698
	T3	6.7	10.7	4.4	8.2	0.241
Oral function	T0	1.8	2.1	2.1	2.2	0.419
	T1	1.9	2.2	1.8	2.1	0.914
	T2	2.1	2.3	1.7	1.9	0.449
	T3	1.9	2.2	1.8	1.9	0.917
Orofacial pain	T0	3.1	2.2	3.4	2.3	0.535
	T1	2.5	2.1	2.4	2.0	0.759
	T2	3.0	2.3	2.0	1.9	0.085
	T3	2.4	2.1	2.3	1.9	0.854
Orofacial appearance	T0	3.2	2.2	3.4	2.1	0.634
	T1	2.7	2.0	2.5	2.0	0.649
	T2	2.6	2.3	2.3	2.0	0.494
	T3	2.5	2.2	2.7	2.2	0.804
Psychosocial impact score	T0	2.0	1.9	2.9	2.0	0.016*
	T1	1.8	1.9	2.3	1.8	0.278
	T2	2.0	1.9	2.2	2.1	0.729
	T3	1.8	1.6	1.4	1.2	0.459

*$p < 0.05$. Equality of variance assumed, two-tailed T-test, SD: Standard deviation.

Table S2. Comparison of the surgically and the non-surgically treated group over the timepoints T0, T1, T2 and T3 with regard to QLQ-C30 scores.

* $p < 0.05$. Equality of variance assumed, two-tailed T-test.

		Surgical treatment		Non-surgical treatment		*p*-value for group difference, - 2-sided
	Timepoint	Mean	SD	Mean	SD	
Global health status	T0	55.1	22.0	49.0	21.7	0.159
	T1	55.8	26.1	44.3	23.1	0.036*
	T2	50.5	25.6	40.6	23.1	0.095
	T3	58.6	17.5	46.5	26.7	0.047*
Functional scales						
Physical functioning	T0	67.9	25.9	52.0	26.2	0.002*
	T1	66.8	27.5	48.0	29.7	0.002*
	T2	66.7	26.9	44.9	29.2	0.001*
	T3	65.1	25.3	58.2	30.5	0.398
Role functioning	T0	61.9	33.9	39.1	38.0	0.001*
	T1	64.5	34.0	39.9	35.5	0.001*
	T2	57.7	33.2	37.7	35.6	0.012*
	T3	60.1	30.9	59.7	37.9	0.966
Emotional functioning	T0	62.9	26.5	57.3	21.8	0.268
	T1	70.8	25.7	57.8	24.9	0.018*
	T2	64.6	27.4	59.4	27.1	0.417
	T3	71.1	21.6	70.8	24.2	0.965
Cognitive functioning	T0	80.3	23.5	74.0	24.7	0.178
	T1	80.4	23.8	67.3	31.9	0.017*
	T2	76.4	25.6	63.0	26.1	0.028*
	T3	79.5	23.1	72.2	23.9	0.321
Social functioning	T0	65.6	32.2	62.0	34.2	0.575
	T1	72.5	31.9	52.4	39.2	0.005*
	T2	70.6	31.4	64.5	38.0	0.428
	T3	72.8	25.6	75.0	33.7	0.794
Symtom scales						
Fatigue	T0	46.5	28.2	59.4	26.2	0.021*
	T1	38.7	30.8	54.0	27.0	0.018*
	T2	47.5	30.4	53.6	25.9	0.376
	T3	44.8	25.4	37.0	27.8	0.336
Nausea and vomiting	T0	8.8	16.1	15.1	23.3	0.074
	T1	9.8	24.6	8.9	17.3	0.862
	T2	7.5	17.3	14.5	26.3	0.125
	T3	7.2	13.6	2.8	9.6	0.278
Pain	T0	43.0	33.6	46.9	35.5	0.569
	T1	31.3	33.3	47.0	37.4	0.032*
	T2	41.0	32.9	44.2	32.8	0.680
	T3	40.3	31.1	27.8	32.0	0.202
Dyspnoea	T0	30.4	31.7	34.4	32.2	0.533
	T1	26.0	32.2	33.3	32.7	0.285

	T2	30.8	33.8	42.4	29.4	0.140
	T3	29.9	31.6	33.3	28.4	0.726
Insomnia	T0	38.1	34.8	45.8	31.4	0.252
	T1	33.3	32.2	45.7	32.2	0.077
	T2	41.5	34.0	26.1	33.3	0.054
	T3	38.7	32.9	30.6	30.0	0.424
Appetite loss	T0	24.4	33.7	26.0	34.6	0.808
	T1	16.2	27.1	32.1	36.8	0.011*
	T2	20.7	32.2	27.5	38.5	0.388
	T3	18.8	25.9	27.8	37.2	0.306
Constipation	T0	16.8	30.2	27.1	77.8	0.238
	T1	12.8	29.7	20.2	29.2	0.241
	T2	18.3	32.3	20.3	29.7	0.791
	T3	17.4	27.8	25.0	32.2	0.395
Diarrhoea	T0	19.4	30.1	14.6	25.3	0.404
	T1	13.5	24.9	6.0	20.4	0.143
	T2	13.3	27.7	14.3	29.0	0.889
	T3	11.4	21.4	13.9	26.4	0.726
Financial difficulties	T0	13.2	25.8	19.8	32.6	0.223
	T1	12.3	25.6	26.2	35.6	0.021*
	T2	15.7	26.2	23.2	38.2	0.273
	T3	17.4	26.2	12.1	16.8	0.520
